# Severe hypothyroidism‐induced rhabdomyolysis: A case report

**DOI:** 10.1002/ccr3.5107

**Published:** 2021-12-07

**Authors:** Mohamed Abdunasser Baghi, Jaseem Sirajudeen, Vamanjore A. Naushad, Khaled S. Alarbi, Nada Benshaban

**Affiliations:** ^1^ Department of General Internal Medicine Hamad Medical Corporation Doha Qatar; ^2^ Department of Critical Care Medicine Tripoli Medical Center Tripoli Libya

**Keywords:** creatine kinase, hypothyroidism, myoglobin, rhabdomyolysis

## Abstract

Hypothyroidism causing rhabdomyolysis is a known but an uncommon entity. Hashimoto's thyroiditis causing rhabdomyolysis in the absence of precipitating factors is even rarer. Here, we report a 42‐year‐old previously healthy man with hypothyroidism due to Hashimoto's thyroiditis complicated by severe rhabdomyolysis and acute kidney injury in the absence of precipitating factors. The diagnosis was based on his clinical presentation and laboratory investigations, and he was successfully treated with intravenous fluid therapy and oral levothyroxine.

## Abstract 2

Rhabdomyolysis can be a rare presentation of hypothyroidism. It is advisable to screen for hypothyroidism in patients with rhabdomyolysis, particularly in those with no obvious cause for raised myogenic enzymes.

## 1 INTRODUCTION

Worldwide, the most common cause of hypothyroidism is iodine deficiency. However, in areas with normal or adequate iodine intake, Hashimoto's thyroiditis remains the most common cause of hypothyroidism, with an estimated annual incidence of 0.3–1.5 cases per 1,000 persons.^1^ Muscular manifestations of hypothyroidism range from muscle weakness, myalgia, muscle cramps with mild to moderately elevated creatine kinase to more severe forms such as Hoffman's syndrome or rhabdomyolysis.^2^ Nevertheless, rhabdomyolysis due to hypothyroidism even in the presence of precipitating factors is rare.^3^ Here, we report an unusual case of rhabdomyolysis due to hypothyroidism without any other associated precipitating factors.

## 2 CASE PRESENTATION

A 42‐year‐old gentleman with no prior medical illness admitted with complaints of generalized muscle pain, dry skin, and mild facial puffiness of eight days duration, associated with choking sensation in his throat. The review of systems was negative for fever, hoarse voice, cold intolerance, hair loss, dysphagia, constipation, weight gain, focal limb weakness, or changes in memory. He denied doing strenuous exercise recently, alcohol consumption, trauma, or recent medication use. There was no family history of autoimmune thyroid diseases.

His vital signs were as following: pulse rate, 65/min (regular); blood pressure, 120/85 mmHg; respiratory rate, 19/min; and oral temperature, 37.1^°^C. Physical examination revealed mild facial puffiness, dry skin, and minimal non‐pitting lower limb edema. A small goiter without tenderness or nodule was found on neck examination. The musculoskeletal examination did not show muscle wasting, hypertrophy, or weakness. Other system examinations were unremarkable.

Laboratory investigations were suggestive of severe hypothyroidism: thyroid‐stimulating hormone (TSH), >100 mIU/ml (normal range <4.35 mIU/L); free T4, <0.5 ng/dl (normal range 11 – 23.3 pmol/L); anti‐thyroid peroxidase antibody titer, >600 IU/ml (normal range <34 IU/ml); and anti‐thyroglobulin antibody (TgAb) titer, 1831 IU/ml (normal range <115). Elevated levels of anti‐thyroid peroxidase antibody and anti‐thyroglobulin antibody titers were suggestive of Hashimoto's thyroiditis. Serum creatine kinase (21,644 U/L, normal range 39–308 U/L) and myoglobin (2,208 ng/ml, normal range 28–72 ng/ml) levels were also raised (Table 1). This was associated with acute kidney injury with mild elevation of serum creatinine (1.44 mg/dl). Urine examination was negative for myoglobinuria or hematuria. The daily urine output was normal (250–300 ml/h).

**TABLE 1:** Results of laboratory investigations on admission and during the follow‐up

Laboratory variables	1st day Admission	4th day Discharge	4th week Follow‐up	6th week Follow‐up	Reference range
White blood cell count (×103/ul)	9	10	8.9	‐	4 – 10
Hemoglobin (gm/dl)	15	15.4	13.3	‐	13.0 – 17.0
Red blood cell count (×106/ul)	5.4	5.4	4.5	‐	4.5 – 5.5
Hematocrit (%)	47.5	48.7	40	‐	40 – 50
Platelet count (×103 /ul)	315	296	297	‐	150 – 400
Alanine aminotransferase (U/L)	127	107	39	24	0 – 41
Aspartate aminotransferase (U/L)	341	266	42	27	0 – 40
Bilirubin T (umol/L)	5	2	5	6	0 – 21
Alkaline phosphatase (U/L)	59	63	76	68	40 – 129
Creatinine (mg/dl)	1.44	1.49	1.2	‐	0.5 – 1.5
Urea (mmol/L)	4.2	4.1	4.9	‐	2.8 – 8.1
Sodium (mmol/L)	140	136	139	‐	135 – 145
Potassium (mmol/L)	4	4.1	5	‐	3.5 – 5.1
Chloride (mmol/L)	103	100	105	‐	95 – 108
Uric acid (umol/L)	527	472	‐	439	200 – 430
Magnesium (mmol/L)	0.8	‐	‐	‐	0.7 – 1.0
Calcium (mmol/L)	2.55	‐	‐	‐	2.12 – 2.60
Creatine kinase (U/L)	21,644	10,969	1,543	685	39 – 308
Myoglobin (ng/ml)	2,208	356	241	127	28 – 72
TSH (mIU/L)	>100	‐	80	34	0.30 – 4.2
FT4 (pmol/L)	<0.5	‐	11	11.8	11 – 23
Anti‐thyroid peroxidase Ab (IU/ml)	>600	‐	‐	‐	0 – 34
Anti‐thyroglobulin Ab (IU/ml)	1,831	‐	‐	‐	0 – 115
HDL‐cholesterol (mmol/L)	1.0	‐	‐	‐	>1.0
LDL‐cholesterol (mmol/L)	2.7	‐	‐	‐	3.3–4.1
Total cholesterol (mmol/L)	5.2	‐	‐	‐	<5.1
Triglyceride (mmol/L)	3.5	‐	‐	‐	<1.7

Abbreviations: FT4, free thyroxine; HDL, high‐density lipoprotein; LDL, low‐density lipoprotein; TSH, thyroid‐stimulating hormone.

Electrocardiogram (ECG) and chest X‐ray were normal. Transthoracic echocardiography (TTE) showed minimal circumferential pericardial effusion without regional wall‐motion abnormalities and with a left ventricular ejection fraction (EF) of 58%. Fiber‐optic (flexible) laryngoscopy was normal.

**FIGURE 1:**
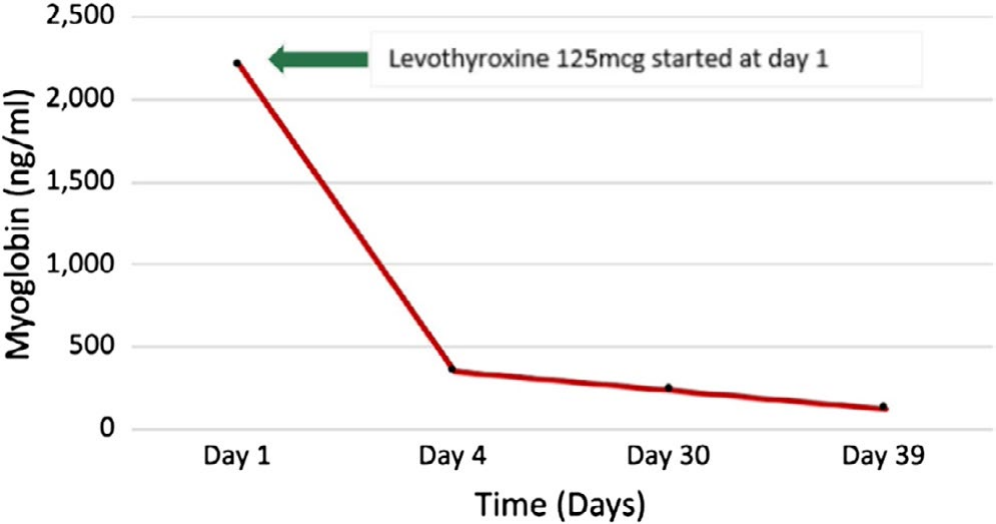
Myoglobin trend over time

**FIGURE 2:**
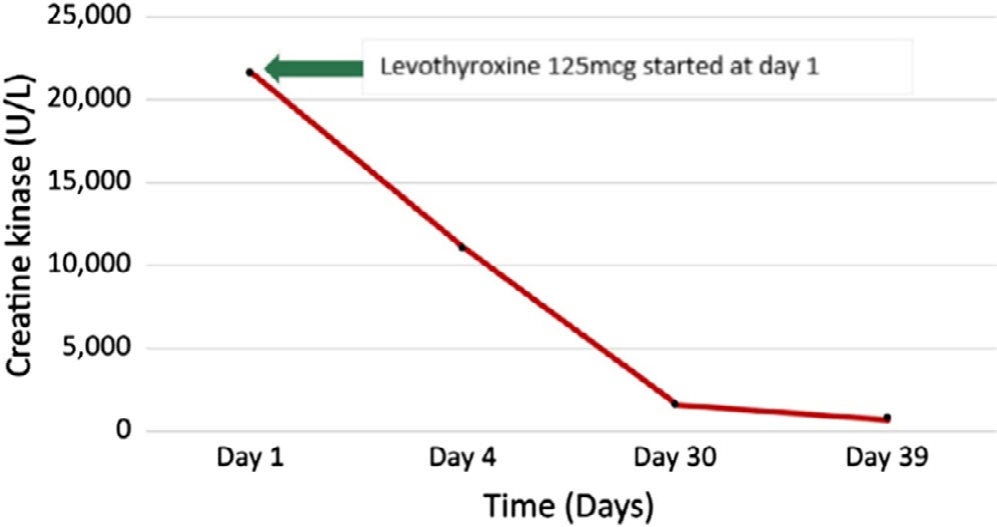
Creatine kinase trend over time

A probable diagnosis of hypothyroidism due to Hashimoto's thyroiditis with rhabdomyolysis was made based on the clinical and laboratory parameters. He was treated with intravenous fluids and was started on oral levothyroxine. His symptoms improved with the treatment, and the levels of CK and myoglobin showed a decreasing trend. He was discharged on Day 4, and on further follow‐up, the muscle enzymes showed a further decreasing trend (Figures 1 and 2) and normalization of renal parameters. Since there was complete resolution of symptoms with the patient returning to his regular day‐to‐day activities, further workup to exclude muscle diseases was not carried out.

## 3 DISCUSSION

Hypothyroidism manifests with a broad spectrum of clinical features. The Involvement of muscle in various forms is frequently found in hypothyroidism.^3^ The muscular symptoms range from stiffness, weakness, myalgia, cramps, pseudohypertrophy and rhabdomyolysis.^2^, ^4^ Rhabdomyolysis is a syndrome characterized by muscle necrosis and the release of intracellular muscle constituents into the circulation.^4^ The causes of rhabdomyolysis can be due to traumatic or non‐traumatic. The various non‐traumatic causes include heat exhaustion, electrolyte imbalance, seizures, endocrine disorders, infections, and heavy exercise.^5^ However, hypothyroidism causing rhabdomyolysis is an infrequent clinical entity and Hashimoto's thyroiditis causing rhabdomyolysis is even rarer, and as per our knowledge, only two cases have been reported so far.^7^, ^8^

Very few cases of hypothyroidism causing rhabdomyolysis have been reported in the literature.^9^ Our patient was diagnosed to have rhabdomyolysis without any apparent cause or risk factors. We could not find a definite cause for rhabdomyolysis in the initial evaluation; hence, hypothyroidism was considered an underlying etiology, which was confirmed by the laboratory investigations. From a review of literature on ten reported hypothyroidism cases causing rhabdomyolysis, it was observed that only four cases had preexisting hypothyroidism when they presented with rhabdomyolysis. In the remaining six cases, hypothyroidism was diagnosed concurrently with rhabdomyolysis.^10^

In cases with rhabdomyolysis due to hypothyroidism, the levels of CK have been reported to be elevated, usually less than ten times the normal range.^7^, ^15^ In the present case, the levels were elevated up to more than 70 times the normal range, which is very unusual in hypothyroidism‐induced rhabdomyolysis. The risk of rhabdomyolysis in hypothyroidism is greater in patients using statins or after rigorous exercise^12^. However, there was no history suggestive of any risk factors in the present case, including statin use or exercise. The pathophysiology of rhabdomyolysis in hypothyroidism is unclear. Various hypotheses have been postulated, such as mitochondrial oxidative metabolism, induction of insulin‐resistant state, and decreased muscle carnitine levels, including autoimmune mechanism.^11^, ^13^ Also, deficiency in thyroxine leads to abnormal glycogenolysis and increased triglyceride turnover, thus leading to the impairment of muscle function by causing a transition from fast‐twitching type 2 muscle fibers to slow‐twitching type 1 fibers, low myosin ATPase activity, and low ATP turnover in the skeletal muscles.^14^

## 4 CONCLUSIONS

Hypothyroidism should be considered as a differential diagnosis of rhabdomyolysis, primarily when no other apparent cause can be found. Early diagnosis and prompt treatment are essential to prevent further serious complications.

## CONFLICT OF INTEREST

All authors declare no conflicts of interest.

## AUTHOR CONTRIBUTIONS

M. Baghi: wrote the manuscript, collected the data, and performed follow‐up. J. Sirajudeen, V. Naushad, K. Alarbi, and N. Benshaban: collected the data.

## ETHICAL APPROVAL

This case was approved by Hamad Medical Corporation's Medical Research Center, and patient consent has been signed and collected in accordance with the journal's patient consent policy.

## Data Availability

Data sharing is not applicable to this article as no datasets were generated or analyzed during the current study.

## References

[1] Vanderpump MP , French JM , Appleton D , Tunbridge WM , Kendall‐Taylor P . The prevalence of hyperprolactinaemia and association with markers of autoimmune thyroid disease in survivors of the Whickham Survey cohort. Clin Endocrinol (Oxf). 1998;48(1):39‐44. 10.1046/j.1365-2265.1998.00343.x 9509066

[2] Khaleeli AA , Griffith DG , Edwards RH . The clinical presentation of hypothyroid myopathy and its relationship to abnormalities in structure and function of skeletal muscle. Clin Endocrinol (Oxf). 1983;19(3):365‐376. 10.1111/j.1365-2265.1983.tb00010.x 6627693

[3] Sindoni A , Rodolico C , Pappalardo MA , Portaro S , Benvenga S . Hypothyroid myopathy: a peculiar clinical presentation of thyroid failure. Review of the literature. Rev Endocr Metab Disord. 2016;17(4):499‐519. 10.1007/s11154-016-9357-0 27154040

[4] Norris FH Jr , Panner BJ . Hypothyroid myopathy. Clinical, electromyographical, and ultrastructural observations. Arch Neurol. 1966;14(6):574‐589. 10.1001/archneur.1966.00470120006002 5935952

[5] Leonetti F , Dussol B , Berland Y . Rhabdomyolyse et insuffisance rénale au cours d'une hypothyroïdie [Rhabdomyolysis and kidney failure in hypothyroidism]. Presse Med. 1992;21(1):31‐32. 1531261

[6] Jain S , Bhargava K , Sawlani KK , Daga MK , Gaiha M . Myoglobinuria and transient acute renal failure in a patient revealing hypothyroidism. J Assoc Physicians India. 1999;47(4):444‐446. 10778535

[7] Jobé J , Corman V , Fumal A , Maertens de Noordhout A , Legros JJ . Le cas clinique du mois. Rhabdomyolyse et hypothyroïdie [Rhabdomyolysis and hypothyroidism]. Rev Med Liege. 2007;62(7–8):484‐486. 17853668

[8] Nikolaidou C , Gouridou E , Ilonidis G , Boudouris G . Acute renal dysfunction in a patient presenting with rhabdomyolysis due to Hypothyroidism attributed to Hashimoto's Disease. Hippokratia. 2010;14(4):281‐283. 21311639 PMC3031325

[9] Gurala D , Rajdev K , Acharya R , Idiculla PS , Habib S , Krzyzak M . Rhabdomyolysis in a young patient due to hypothyroidism without any precipitating factor. Case Rep Endocrinol. 2019;2019:1‐3. 10.1155/2019/4210431 PMC6914874 31885945

[10] Salehi N , Agoston E , Munir I , Thompson GJ . Rhabdomyolysis in a patient with severe hypothyroidism. Am J Case Rep. 2017;18:912‐918. 10.12659/AJCR.904691 28827517 PMC5574522

[11] Monzani F , Caraccio N , Siciliano G , Manca L , Murri L , Ferrannini E . Clinical and biochemical features of muscle dysfunction in subclinical hypothyroidism. J Clin Endocrinol Metab. 1997;82(10):3315‐3318. 10.1210/jcem.82.10.4296 9329360

[12] Macleod A , Siddique H , Bashir A , Moulik P , Beshr A . Rhabdomyolysis and acute renal failure due to hypothyroidism. Endocr Abstr. 2008;15:P384.

[13] Mouzouri H , El Omri N , Sekkach Y , et al. Rhabdomyolyse sévère révélant une myopathie hypothyroïdienne d'origine auto‐immune A propos de deux cas [Severe rhabdomyolysis revealing a myopathy linked to autoimmune hypothyroidism]. Ann Endocrinol (Paris). 2009;70(1):83‐86. 10.1016/j.ando.2008.05.001 18603225

[14] Kisakol G , Tunc R , Kaya A . Rhabdomyolysis in a patient with hypothyroidism. Endocr J. 2003;50(2):221‐223. 10.1507/endocrj.50.221 12803243

[15] Bosch X , Poch E , Grau JM . Rhabdomyolysis and acute kidney injury. N Engl J Med. 2009;361(1):62‐72. 10.1056/NEJMra0801327 19571284

